# Weak effect of urbanization on bdelloid rotifers living in lichens

**DOI:** 10.1098/rsos.231978

**Published:** 2024-04-17

**Authors:** Rebecca Partemi, Nicolas Debortoli, Alejandro Martínez, Lyudmila Kamburska, Caroline Souffreau, Hans Matheve, Pieter Vantieghem, Luc De Meester, Karine Van Doninck, Thomas Merckx, Diego Fontaneto

**Affiliations:** ^1^ Department of Chemical and Geological Sciences, Modena and Reggio-Emilia University, Modena 41125, Italy; ^2^ National Research Council of Italy (CNR), Water Research Institute (IRSA), Verbania Pallanza, 28922, Italy; ^3^ Namur Molecular Tech, CHU UCL Namur, Yvoir 5530, Belgium; ^4^ National Biodiversity Future Center (NBFC), Palermo 90133, Italy; ^5^ Laboratory of Freshwater Ecology, Evolution & Conservation, KU Leuven, Charles Deberiotstraat 32, Leuven 3000, Belgium; ^6^ Terrestrial Ecology Unit, Department of Biology, Ghent University, Gent 9000, Belgium; ^7^ Leibniz Institut für Gewässerökologie und Binnenfischerei (IGB), Berlin 12587, Germany; ^8^ Institute of Biology, Freie Universität Berlin, Berlin 14195, Germany; ^9^ Research Unit in Molecular Biology and Evolution, DBO, Université libre de Bruxelles (ULB), Brussels 1050, Belgium; ^10^ WILD, Biology Department, Vrije Universiteit Brussel (VUB), Brussels 1050, Belgium

**Keywords:** anthropogenic effects, biodiversity, community ecology, Rotifera Bdelloidea, urban ecology

## Abstract

Human activities have an overwhelming impact on the natural environment, leading to a deep biodiversity crisis whose effects range from genes to ecosystems. Here, we analysed the effect of such anthropogenic impacts on bdelloid rotifers (Rotifera Bdelloidea), for whom these effects are poorly understood. We targeted bdelloid rotifers living in lichen patches across urbanization gradients in Flanders and Brussels (Belgium). Urbanization was measured as the percentage of built-up area (BU) across different spatial scales, at circles from 50 to 3200 m of radius around the lichen. Urbanization effects on biodiversity were assessed on abundance, species richness and community-weighted mean body size of bdelloid rotifers, as well as on genetic diversity of a mitochondrial marker (cytochrome *c* oxidase subunit I) of one of the most common and widespread bdelloid species, *Adineta vaga*. Overall, no negative effect of urbanization was found at any diversity level and any spatial scale. Counterintuitively, the BU area quantified at the largest spatial scale had a positive effect on abundance. These results leave open the question of whether negative effects of urbanization are present for bdelloid rotifers, if they are mediated by other unexplored drivers, or if such effects are only visible at even larger spatial scales.

## 1. Introduction

The conversion of natural and rural areas into urban areas, called urbanization, has the potential to affect patterns and processes of biodiversity throughout the planet [1,2]. Urbanization generally brings habitat loss and pollution, which are among the most severe threats that biodiversity may face, translating into shrinking population sizes and species loss across communities [3–5].

Negative consequences of urbanization have been documented for populations of various animal species [6], even if some synanthropic species may also take advantage of the new conditions [7,8]. However, much less is known about the effect of urbanization on microscopic animals, which could be affected in different and unpredictable ways [9,10]. We know that numerous invertebrate taxa, including microscopic ones, may respond negatively to anthropogenic disturbance in general and to urbanization in particular [6,11]. Yet, we lack a robust quantification of the strength and direction of those responses to understand how they connect to different functional traits [12].

Among the most common and ubiquitous microscopic animals that can be found in any habitat, including highly urbanized areas, are the bdelloid rotifers [13]. Some species of bdelloid rotifers, and other microscopic animals, exhibit large distribution ranges across the world, potentially owing to their highly resistant dormant stages that allow long-range passive dispersal even between continents [14]. Yet, even if some bdelloid rotifer species may have wide distributions, different species are known to be adapted to different environmental conditions [15,16] as also observed in monogonont rotifers [17]. We can thus reliably assume that anthropogenic effects could lead to detectable species decline and/or loss for bdelloid rotifers.

Generally, bdelloid rotifers thrive in any habitat where water becomes available, even for short periods, owing to their peculiar ability to survive complete desiccation at any life cycle stage. Some rotifer species also occur in so-called extreme habitats, both of natural origin, such as the Atacama desert [16], high mountains [18], the inhospitable polar regions [19] and of anthropogenic origin, such as mine lakes [20] and heavy metal polluted waters [21]. However, the few species that can indeed occur in anthropogenically modified environments are those that naturally occur in similar habitats, for example, with comparable levels of low pH or high load of heavy metals [22,23]. Even if rotifers as a clade can be found almost anywhere and in any habitat, different rotifer species have species-specific ecological tolerances to contrasting environmental conditions. Hence, we hypothesize that different rotifer communities should be found in natural, rural and urban areas, with different species exhibiting species-specific traits that enable them to thrive in their respective habitats. Yet, previous studies analysing responses of rotifers to urbanization did not report a clear pattern [5,12,24,25].

Here, we address this hypothesis by testing whether patterns of diversity in bdelloid rotifers reflect a gradient of anthropogenic pressure connected to differential levels of urbanization measured at different spatial scales. In order to minimize potential confounding factors, we selected communities of bdelloid rotifers living in lichens of the same genus, collected in a short period of time. We explored diversity patterns of bdelloid rotifers at the community level, such as species richness and community composition, but also patterns at the genetic level, targeting metrics of population genetic diversity of one of the most common species in the samples.

## 2. Material and methods

### 2.1. Sampling design

Sampling was performed in Belgium, in a polygon of 8140 km^2^, encompassing the cities of Brussels (Brussels region), Antwerp, Leuven and Ghent (Flanders region). The area is densely populated (average human population density of Flanders: 480 inhabitants km^-^², Brussels: 5700 inhabitants km^-^² [26]), and is composed of urban areas embedded within a semi-natural and agricultural matrix. Because urbanization encompasses a range of factors that alter the physical environment and landscape characteristics, we defined the proportion of built-up area (BU) as a proxy for urbanization. The proportion of BU area was assessed with a GIS software (ArcMap 10) using an object-oriented reference map of Flanders as a vectorial layer [27]. This layer included the precise contours of all buildings, while roads and parking infrastructures were excluded. To account for the potential effects of urbanization at the landscape scale, we selected 27 plots (i.e. squares of 3 km × 3 km) across a grid encompassing the whole sampling area (electronic supplementary material, figure S1). Nine plots were located in low-urbanized areas (low: 0–3% BU), nine plots in areas with intermediate urbanization (intermediate: 5–10% BU) and nine in high-urbanized areas (high: >15% BU). Given that only buildings were considered for the calculation of BU areas, values of 15% corresponded to highly urbanized areas, that is, mostly city centres. We first selected the nine plots within the highest proportion of BU area, so that they were approximately equidistant from each other within the study area. Next, plots of the intermediate and lowest urbanization categories were selected within 10–25 km from the highest urbanized plots. This strategy resulted in an evenly spread selection of plots within the same urbanization category across the study area and ensured minimal spatial autocorrelation of plot urbanization levels. Across plots, % BU was positively correlated with the amount of other impervious substrates such as roads and artificial constructions (bridges, viaducts, etc.) (Pearson *r* = 0.94; *p* < 0.0001) and negatively correlated with the area of semi-natural habitat (*r* = −0.85; *p* < 0.0001) [12], thus representing a reliable proxy of urbanization. To investigate the effects of local-scale urbanization, each plot was then divided into a grid of local subplots of 200 m × 200 m, which were classified into urbanization categories using identical % BU thresholds as used at the plot level. Within each plot, we then selected one subplot of each urbanization category (i.e. low, intermediate and high). This selection was meant to be random, with minimum deviations owing to constraints imposed by accessibility and the permission to sample.

The sampling design resulted in a total of 81 sampling sites (i.e. nine plots × three landscape-scale urbanization levels × three local-scale urbanization levels (see fig. 1 in [12])), covering the entire range of urbanization levels available in this part of Belgium. The design guaranteed that urbanization at landscape and local scales should be uncorrelated and, hence, their effects, as well as their interaction, could be reliably tested simultaneously.

For a more detailed description of the sampling scheme for bdelloid rotifers, we refer to Merckx *et al*. [12] and Piano *et al*. [5].

### 2.2. Biodiversity metrics

Communities of bdelloid rotifers were sampled by collecting one lichen patch in each subplot. We selected lichens of the genus *Xanthoria*, for which bdelloid rotifer communities have been previously studied in Europe [15]. Lichens of the genus *Xanthoria* are among the most abundant in urban and rural areas, apparently unaffected by urbanization levels [28]. Sampling was performed in June and July 2013. Suitable *Xanthoria* patches could be found in all but two subplots: the total sample size is thus 79 lichens and not 81. The two missing subplots were not from highly urbanized ones: one was from a subplot of intermediate urbanization within a plot of low urbanization and the other was from a subplot of low urbanization within a plot of high urbanization. No apparent bias was evident in the two missing plots skewing the data towards or against high urbanization.

The selection of the lichen was haphazard: the first suitable lichen patch encountered in each subplot on a natural substrate (e.g. tree trunk and branch) was collected. Dry lichen thalli between 5 and 10 cm² were cut from the substrate with a knife and kept in zip lock bags. For each lichen sample, an area of 2.5 cm^2^ was hydrated with distilled water in a plastic petri dish to identify bdelloid species, and another area of 2.5 cm^2^ was used later to extract the animals to be used for the genetic analyses of the focal species.

All active bdelloid rotifers that recovered from dormancy within the four hours following hydration in the laboratory were sorted and identified to species level according to [29]. Previous studies on bdelloid rotifers in these lichens [15] revealed that animals start recovering between 10 and 40 min after hydration of the sample and that no more additional bdelloid rotifers usually recover after 4 h. The very few (from none to two) dormant stages still found in the sample that did not recover after that time were considered dead, impossible to identify at any taxonomic rank, and excluded from the analyses.

All living bdelloids were isolated, counted and identified to species level to obtain data on (i) abundance and (ii) species richness. The other descriptor of community-level diversity was (iii) community-level body size [12], calculated as community-weighted mean body size, which is the average of the species-specific body length for all sampled species in a community, weighted by species abundance in the community. Measurements of body length for the observed species, obtained from the observed animals, are reported in Merckx *et al*. [12].

The most common and abundant species in all samples, *Adineta vaga*, was selected to obtain metrics of genetic diversity. DNA was extracted from each of the individuals found in the part of each lichen patch that was not used for the morphological identifications but to extract the focal species. We amplified the barcoding fragment of the mitochondrial marker cytochrome *c* oxidase subunit I (COI), using Folmer primers with optimized protocols for this species [30]. COI sequences were trimmed to a total common length of 605 bp and aligned using default settings in MAFFT v7 [31], confirming correct amino acid translation. Species identity was confirmed through online BLAST searches [32]. The abundance of haplotypes of *A. vaga* for each population of the species in each lichen patch was used as a metric of genetic diversity.

Given the aim of this study, the chosen descriptors of urbanization levels for our statistical analyses were the different proportions of BU area at different spatial scales, measured from the coordinates of the lichen including circles of radius from 50 m (BU50) to 3200 m (BU3200). Proportions of BU area at different spatial scales (namely, BU50, BU100, BU200, BU400, BU800, BU1600 and BU3200) have a nested structure making them not independent: for example, a radius with a high proportion of BU area at one level, for example, 100 m, will most likely also have a high proportion at a similar spatial level, for example, 200 m. We accounted for such an effect in the statistical models (see later). Given the structure of the sampling design, we are confident that autocorrelation owing to the nested structure will not be present for pairs of radii at larger distances (e.g. 50 and 400 m).

### 2.3. Drivers of community-level diversity

We first checked for the correlation between levels of urbanization at different spatial scales, calculated as proportions of BU surface in areas at different radii, from 50 to 3200 m, using the R [33] package psych v2.0.12 [34], in order to minimize redundancy and use only a set of non-correlated variables to try and explain bdelloid diversity patterns. Using a threshold of Pearson’s *r* = 0.80, a subset of proportions of BU area at three uncorrelated spatial scales (namely, BU50, BU200 and BU800) was retained for the following analyses (electronic supplementary material, figure S2).

The first set of hypotheses we tested included whether urbanization could affect the abundance, species richness and community body size of individuals we found. The model included the total number of individuals per patch, or the number of species, or the average community body size (response variable) as a function of the three uncorrelated metrics of urbanization of different spatial scales (BU50, BU200 and BU800), in addition to the substrate of the lichen (artificial or tree bark). Abundance of individuals was included as an additional predictor for the models on species richness and community body size.

These three main models were performed considering the potential existence of spatial effects by testing various correlation structures (spherical, linear, ratio, Gaussian and exponential) in spatially explicit generalized least square models in the R package nlme v 3.1-162 [35]. The model with the significantly lowest Akaike Information Criterion (AIC) was then selected to inspect the output. In cases when no spatial structure was needed in the model, we then used simpler linear models (LM) with the same structure of response and predictor variables. Model assumptions (e.g. normality of residuals, homogeneity of variance, influential observations, collinearity, etc.) were checked with the R package performance v0.10.2 [36] and response variables were transformed when necessary (e.g. abundance with log values) to improve model fit. Partial *R* ^2^ for each predictor was calculated with the R package asbio v1.9-2 [37].

We also tested the effect of urbanization, again controlling for substrate type, on community composition using Moran’s eigenvector mapping (MEM), to assess the unique and combined effect of spatial structure and environmental variables in shaping the distribution of haplotypes. To do so, we used the R package adespatial v0.3-21 [38]. To then test the detailed effects of urbanization (controlling for substrate type) on the occurrence of the different species in the lichens, we used a model-based approach to the analysis of multivariate abundance data [39]. The model included the multivariate abundance data of the species (response variable) as a function of the proportion of BU area at the three uncorrelated spatial scales (BU50, BU200 and BU800), in addition to the type of substrate in the R package mvabund v4.2.1 [39].

The R script for the analyses at the community level is provided as supplementary material (electronic supplementary material, file 03: speedy_bdello.R), together with the dataset (electronic supplementary material, file 04: speedy_bdello.csv).

### 2.4. Drivers of genetic diversity

The number of haplotypes of the focal species *A. vaga* was analysed with statistical models following what was done for species richness using as predictors the three uncorrelated metrics of urbanization of different spatial scales (BU50, BU200 and BU800), the substrate of the lichen (artificial or tree bark), in addition to the number of sequenced animals as a confounding factor to account for sample size.

To test for the effect of urbanization in structuring genetic differences between populations, Hedrick’s G_ST_ [40,41] was used as a metric of genetic diversity between populations. It was calculated with the R package mmod v1.3.3 [42], handling DNA data with packages apex v1.0.4 [43] and adegenet v2.1.3 [44]. To assess the relationship between genetic diversity and the level of urbanization, accounting for the effect of geographical distances between lichens, we used Mantel tests with the R package vegan v2.6-4 [45]. As a matrix of geographical distances, we calculated the geodesic distance in km between lichen patches with the R package geodist v0.0.7 [46]; as a matrix of ecological differences, we calculated the Gower distances using the three selected spatial scales of BU area with the R package ecodist v2.0.9 [47]. We then also used a permutational multivariate analysis of variance using distance matrices (adonis) to test how much of the genetic diversity between populations (GST differentiation) could be affected individually by the three uncorrelated metrics of urbanization of different spatial scales and the substrate of the lichen, using the R package vegan.

The R script for the analyses of genetic diversity is provided as supplementary material (electronic supplementary material, file 05: Speedy_COI.R), together with the datasets (electronic supplementary material, file 06: Speedy_COI.fas; file 07: Speedy_COI.csv and file 08: Speedy_COI_data.csv).

## 3. Results

### 3.1. Bdelloid diversity

Out of the 79 lichen patches, we found bdelloids in 75 lichens and counted and identified 4936 individuals of bdelloid rotifers belonging to 21 species (GBIF dataset: https://www.gbif.org/dataset/c7b00a37-f7e5-469a-b504-3a9d11757fd2) [48]. Abundance of individuals was up to 145 animals cm^-3^, with average ±s.d.: 25 ± 30 (median = 14.5). Richness of bdelloid rotifers in each lichen patch was up to six species, on average 2.5 ± 1.4 (median = 2). The most abundant species was *A. vaga*, with 742 individuals in 32 lichen patches, followed by *Mniobia russeola*, with 710 individuals in 21 lichen patches and *Macrotrachela ehrenbergi* with 639 individuals in 33 lichen patches.

The three metrics of diversity (i.e. abundance of individuals, species richness and community body size) were not correlated (Pearson’s *r* from 0.20 to 0.64) and captured different aspects of biological diversity in the bdelloid dataset.

**Figure 1.**
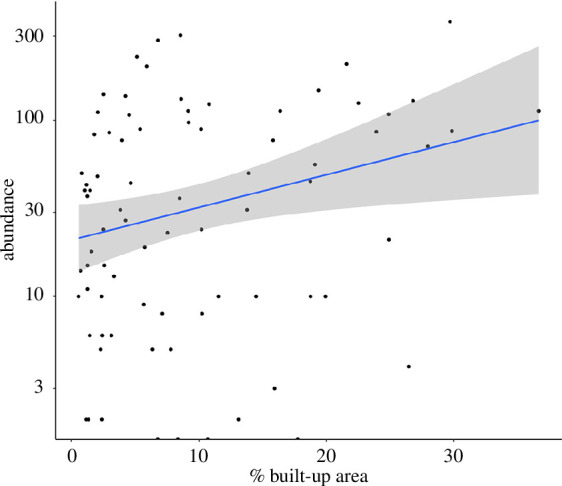
Effect of urbanization (percentage of BU area) on the abundance (number of individuals in 2.5 cm^2^ of lichen) of bdelloid rotifers in lichen patches at 800 m radius. The y-axis is in the log scale. The trend line is depicted with the 95% confidence interval.

The model on abundance of individuals was not affected by spatial autocorrelation (AIC non-spatial model = 308.6, d.f. = 6; AIC best spatial model, Gaussian = 310.3, d.f. = 8). Thus, a simple model not including any spatial structure was used to assess the effect of urbanization: only the proportion of BU area in the largest tested radius, 800 m, significantly explained abundance of bdelloids and it had a positive effect (table 1; figure 1).

**Table 1.** Output of the non-spatially explicit LM to explain abundance of bdelloids (in the log scale) as a function of the proportion of BU area at circles of 50, 200 and 800 m radii around the lichen, and type of substrate for the lichen (artificial or tree bark). (Model estimates with s.e., *t*-value, *p*-value and partial *R* ^2^ are reported. Bold *p*-values highlight significant predictors, below the 0.05 threshold.)

predictor	estimate	s.e.	*t*	*p*	partial *R* ^2^
(intercept)	3.10	0.27	11.5	< 0.0001	
BU50	0.01	0.03	0.4	0.670	0.0025
BU200	− 0.04	0.03	− 1.1	0.273	0.0162
BU800	0.06	0.03	2.3	**0.025**	0.0663
substrate	− 0.42	0.41	− 1.0	0.310	0.0139

**Figure 2.**
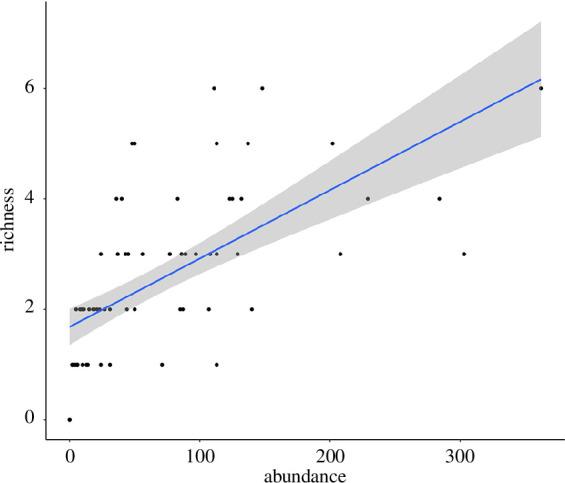
Effect of abundance of individuals on species richness of bdelloid rotifers in the sampled lichen patches. The trend line is depicted with the 95% confidence interval.

The model on richness was not affected by spatial autocorrelation (AIC non-spatial model = 282.5, d.f. = 7; AIC best spatial model, ratio = 283.8, d.f. = 9) and none of the predictors had any effect, whereas richness was clearly explained by abundance of individuals (table 2; figure 2).

**Table 2.** Output of the non-spatially explicit LM to explain richness of bdelloids as a function of the proportion of BU area at circles of 50, 200 and 800 m radii around the lichen, type of substrate for the lichen (artificial or tree bark) and abundance of individuals. (Model estimates with s.e., *t*-value, *p-*value and partial *R* ^2^ are reported. Bold *p*-values highlight significant predictors, below the 0.05 threshold.)

predictor	estimate	s.e.	*t*	*p*	partial *R* ^2^
(intercept)	1.89	0.22	8.4	< 0.0001	
BU50	− 0.02	0.02	− 0.9	0.350	0.0119
BU200	0.01	0.03	0.4	0.667	0.0025
BU800	− 0.01	0.02	− 0.7	0.500	0.0063
substrate	− 0.26	0.32	− 0.8	0.417	0.0090
abundance	0.01	0.01	6.9	**< 0.0001**	0.3999

The model on community body size was not affected by spatial autocorrelation (AIC non-spatial model = 1001.1, d.f. = 7; AIC best spatial model, exponential = 1001.7, d.f. = 9). None of the predictors explained the community body size of bdelloids in lichens (electronic supplementary material, table S1).

Community composition was more strongly explained by spatial patterns than by urbanization effects: MEM revealed that spatial structure alone explained 19% of the variability in species composition, with an additional 1% shared with environmental variables (urbanization and substrate), and environmental variables alone adding only 1% (electronic supplementary material, figure S3). In accordance with the poor explanatory power of the degree of urbanization on community composition supported by MEM, the multivariate abundance of species did not reveal any significant effects either (electronic supplementary material, table S2).

### 3.2. Genetic diversity of the focal species *Adineta vaga*

The focal species *A. vaga* was found in 32 of the lichen patches and we obtained COI sequences for 267 animals from 29 lichen patches, providing 40 haplotypes. All haplotypes had a best BLAST hit to already sequenced animals from the *A. vaga* species complex, from Belgium, Turkey and the UK (electronic supplementary material, table S3). One to four haplotypes were found in each population, on average 2.0 ± 1.3 and median = 2. Each haplotype was found from one to nine populations (electronic supplementary material, table S3), on average 1.6 ± 1.6 and median = 1.

The model on haplotype richness was not affected by spatial autocorrelation (AIC non-spatial model = 136.9, d.f. = 7; AIC best spatial model, Gaussian = 138.8, d.f. = 9), and none of the predictors had any effect, not even the potential confounding factor of the number of sequenced animals (table 3).

**Table 3.** Output of the non-spatially explicit LM to explain haplotype richness of the focal species *A. vaga* as a function of the proportion of BU area at circles of 50, 200 and 800 m radii around the lichen, type of substrate for the lichen (artificial or tree bark) and number of sequenced individuals. (Model estimates with s.e., *t*-value, *p*-value and partial *R* ^2^ are reported.)

predictor	estimate	s.e.	*t*	*p*-value	partial *R* ^2^
(intercept)	2.90	0.49	5.9	< 0.0001	
BU50	0.03	0.04	0.6	0.535	0.0150
BU200	0.01	0.05	0.1	0.915	0.0004
BU800	− 0.02	0.03	− 0.5	0.587	0.0115
substrate	− 1.43	0.85	− 1.7	0.107	0.0969
number of individuals	0.07	0.03	2.0	0.056	0.1331

Mantel tests revealed no correlation of genetic diversity with geographical distance (*r* = 0.025, *p* = 0.358) or with environmental distance between lichen patches (*r* = 0.161, *p* = 0.072). A marginally significant effect on differences in genetic diversity was found for the proportion of BU area at the smallest spatial scale (table 4).

**Table 4.** Output of the permutational multivariate analysis of variance using distance matrices (adonis) to explain genetic diversity between populations of *A. vaga* (GST differentiation) as a function of the levels of urbanization at the three selected spatial scales, considering also substrate type. (d.f., *F*, *R* ^2^ and *p*-values are reported. Bold *p*-values highlight significant predictors, below the 0.05 threshold.)

predictor	d.f.	*F*	*R* ^2^	*p*
BU50	1	2.21	0.0755	**0.046**
BU200	1	1.05	0.0359	0.454
BU800	1	1.05	0.0357	0.451
substrate	1	0.98	0.0336	0.551
residuals	24		0.819	

## 4. Discussion

The first striking result of our survey of bdelloid rotifer diversity in *Xanthoria* lichens across gradients of urbanization is that urbanization itself does not seem to negatively affect the biodiversity of bdelloid rotifers at any level. One of the potential explanations for such an unexpected result could be the fact that the study area (Brussels and the central part of Flanders) does not have truly pristine or unaffected natural areas and is overall degraded in its environmental quality [49]. Even sampling sites that we consider from natural and rural areas with low levels of urbanization in Belgium have indeed already been heavily affected for decades by anthropogenic activities such as atmospheric pollution [50]. Taxa responding to their environment at large spatial scales may also simply be lacking small areas with a low degree of urbanization, not truly reflecting a low degree of anthropogenic influence. If, for example, we assume a potential negative direct effect of air pollution on bdelloids and other microscopic invertebrates [51,52] or also indirectly because of the effects such pollution has on lichens [53], biodiversity effects may not be seen within the small scale of Belgium. The effects of air pollution, or other similar drivers, happen at much larger spatial scales [54]. Therefore, much larger spatial scales or areas with stronger gradients of urbanization may need to be analysed to detect urbanization effects on bdelloid rotifer communities.

One way to test this hypothesis could be to compare the observed richness with that known from other European areas with lower levels of urbanization. Species richness in each lichen patch in the study area in Belgium was on average (±s.d.) 2.5 ± 1.4, with a maximum of six species. This is a lower number than what was found in similar surveys of lichen-dwelling bdelloid rotifers (including those from *Xanthoria* lichens) from less densely urbanized areas, such as in Scandinavia, where lichen patches hosted on average 5.5 ± 2.0 species, up to a maximum of 11 [15]. Other studies provided comparable or even lower richness than the one observed in Belgium: 3.0 ± 1.1 in Anatolia, Turkey [55] , 1.7 ± 0.5 in the eastern part of Turkey [56] and 1.6 ± 0.2 in high mountains in the Italian Alps [57]. These areas in Turkey and Italy are all with lower or even much lower levels of urbanization than the study area in Belgium [58]. The richness of bdelloid rotifers in each lichen patch in the study area in Belgium seems within the known range of variability in Europe, regardless of anthropogenic impacts and urbanization levels. However, species richness in each lichen patch is rather low and with a large variability, making it hard to capture any significant signal. In addition, too little community-level data are available for lichen-dwelling bdelloids to allow any support for the hypothesis that no effects were visible because Belgium could be considered heavily impacted by anthropogenic activities, even in rural and in so-called natural areas. Other hypotheses need to be explored to understand the observed lack of detrimental effect of urbanization on lichen-dwelling rotifers in Belgium.

An alternative explanation of the lack of effect of urbanization on bdelloids could be identified in the ability that bdelloids have to enter a dormant stage, similar to dormancy, in response to environmental stress. Such ability has been studied in relation to lack of oxygen, lack of food, change in pH, etc [59,60]; however, not yet in relation to environmental pollution. Thus, we can only speculate that bdelloids may enter dormancy to survive the pollution peaks owing to urbanization and later recover when conditions improve, with a process that may mask any detrimental effect of urbanization on environmental quality.

If anything, a positive effect of urbanization could be seen on bdelloid abundance at a large spatial scale, that is, at the 800 m radius scale (figure 1). To check that the significant result at 800 m was not a spurious one, we performed univariate models for each of the radii from 50 to 3200 m, and indeed all the radii above 800 m revealed a positive effect of urbanization on abundance (electronic supplementary material, table S4). Repeating the selection of uncorrelated radii starting from the largest (BU3200) and not the smallest radius (BU50), the radii at BU100, BU400 and BU3200 were retained for the analyses that revealed the same qualitative effects of the reported models using BU50, BU200 and BU800 (results not shown). However, urbanization at large scales had relatively low R^2^ (table 1; electronic supplementary material, table S4), making the inference not strongly supported. Yet, a potential explanation for the positive effect of urbanization on abundance could be identified in a stronger detrimental effect of urbanization on potential predators and competitors of bdelloids (e.g. mites [9,61]) than on bdelloids themselves. Bdelloids are known to be able to win the arms race against fungal parasites by occupying habitats and areas where such fungal parasites find suboptimal conditions [62,63]. Thus, if predators and competitors of bdelloids (e.g. tardigrades, nematodes, mites, etc.) disappear at levels of anthropogenic impacts that do not affect bdelloids, the possibility for bdelloids to reach higher abundances at higher levels of urbanization could make sense. Bdelloids are indeed known to thrive and reach high abundances in habitats and areas that are considered ‘extreme’ for other animals, like the polar deserts, where they are among the few forms of animal life, without predators and with almost no competitors [19,64,65].

The other, though barely significant, effect found for urbanization at the small scale of 50 m affecting genetic diversity in the focal species (table 4), is too marginal to be considered reliable. Thus, no clear evidence seems to exist for a negative effect of urbanization on the diversity of lichen-dwelling bdelloid rotifers. The overall lack of urbanization effects on bdelloids living in *Xanthoria* lichens is in line with previous analyses on the same dataset: community-based body-size shifts were demonstrated for several groups of animals in Belgium, but not for bdelloid rotifers, whose metrics remained unaffected by urbanization [12]. On the other hand, Piano *et al*. [5] identified urbanization as a potential driver of differences in community composition on bdelloid rotifers. Yet, such effects were visible only by partitioning richness components of beta diversity in a way that is difficult to compare with the biodiversity metrics used in this study.

Previous studies on the effect of urbanization on other invertebrates revealed that poorly dispersive species tend to disappear more frequently by local extinction processes owing to urbanization, for instance, favouring the dominance of highly dispersive species in moths and ground beetles [66,67]. Lichen-dwelling bdelloids are all highly dispersed passively by the wind when in a dormant desiccated state [14,62]; thus, differential dispersal capabilities between species cannot be a potential driver of differences in bdelloid diversity owing to urbanization.

A decline in abundance caused by urbanization has been highlighted as a potential driver of local extinction [5]. Yet, what we found for lichen-dwelling bdelloid rotifers in Belgium is that abundances are not negatively affected by urbanization, neither in the overall community nor in the single species abundance-based multivariate analyses. Effects of urbanization were found to act at the very local scale of a few tens of metres in freshwater zooplankton [68], but the proportion of BU area at the local scale had no effect on bdelloids.

In summary, we found that bdelloid rotifers may not respond to urbanization or provide ambiguous evidence of effects. Surely, if urbanization is so strong as to remove lichens, the preferred habitat for the lichen-dwelling bdelloid rotifers disappears and they can become locally extinct. Yet, with the current level of urbanization in Flanders, lichens are still able to persist and no clear detrimental effect was found for bdelloid rotifers, for any of the metrics of biodiversity we analysed. It could also be that urbanization has a clear effect on the occurrence and the surface area of lichens, but less so on the diversity of rotifers within each lichen. Such a site-selection bias is known to potentially affect biodiversity analyses [69,70]. Indeed, we could not find any suitable lichen habitat in two of the subplots; these two subplots were not in the most heavily urbanized ones, suggesting that, potentially, no site-selection bias was confounding the effects of urbanization levels. Yet, we cannot exclude such site-selection bias.

Responses of microscopic animals, like rotifers and meiofauna in general, to anthropogenic disturbance, are known to depend on the environmental context in which the disturbance occurs, on the scales at which responses are observed, and on the extent to which the disturbance creates novel environments that differ from those to which the organisms are adapted [71]. Thus, lichen-dwelling bdelloid rotifers may be highly resilient to anthropogenic disturbance owing to urbanization, preventing us from clearly identifying negative effects.

## Data Availability

Occurrence data of bdelloids in the lichen patches is available as a GBIF dataset [48] COI sequences from *A. vaga* are deposited in GenBank with accession numbers OR121101–OR121147, OR121151–OR121260, OR121262–OR121288, OR121291–OR121327 and OR121329–OR121374. All data produced from this study are provided as electronic supplementary material, files 04, 06, 07, 08 and 12 [72].
